# The Effects of Theory-Based Educational Intervention and WhatsApp Follow-up on Papanicolaou Smear Uptake Among Postnatal Women in Malaysia: Randomized Controlled Trial

**DOI:** 10.2196/32089

**Published:** 2022-06-27

**Authors:** Zaahirah Mohammad, Norliza Ahmad, Anisah Baharom

**Affiliations:** 1 Department of Community Health Faculty of Medicine and Health Sciences Universiti Putra Malaysia Serdang Malaysia

**Keywords:** uterine cervical neoplasms, Papanicolaou test, psychological theory, self-efficacy, social media, health knowledge, attitude, practice, Malaysia

## Abstract

**Background:**

Despite the availability and accessibility of free Papanicolaou (Pap) smear as a screening tool for cervical cancer, the uptake of Pap smear in Malaysia has not changed in the last 15 years. Previous studies have shown that the high uptake of Pap smear reduces the mortality rate of patients with cervical cancer. The low uptake of Pap smear is multifactorial, and the problem could be minimized through the use of mobile technologies. Nevertheless, most intervention studies focused on individual factors, while other important aspects such as mobile technologies, especially WhatsApp, have not been investigated yet.

**Objective:**

This study aims to determine the effects of a theory-based educational intervention and WhatsApp follow-up (Pap smear uptake [PSU] intervention) in improving PSU among postnatal women in Seremban, Negeri Sembilan, Malaysia.

**Methods:**

A 2-arm, parallel single-blind cluster randomized controlled trial was conducted among postpartum women from the Seremban district. Twelve health clinics were randomly assigned to the intervention and control groups. At baseline, both groups received a self-administered questionnaire. The intervention group received standard care and PSU intervention delivered by a researcher. This 2-stage intervention module was developed based on Social Cognitive Theory, where the first stage was conducted face-to-face and the second stage included a WhatsApp follow-up. The control group received standard care. Participants were observed immediately and at 4, 8, and 12 weeks after the intervention. The primary endpoint was PSU, whereas the secondary endpoints were knowledge, attitude, and self-efficacy scores for Pap smear screening self-assessed using a Google Forms questionnaire. A generalized mixed model was used to determine the effectiveness of the intervention. All data were analyzed using IBM SPSS (version 25), and P value of .05 was considered statistically significant.

**Results:**

We analyzed 401 women, of whom 76 (response rate: 325/401, 81%) had withdrawn because of the COVID-19 pandemic, with a total of 162 respondents in the intervention group and 163 respondents in the control group. The proportion of Pap smears at the 12-week follow-up was 67.9% (110/162) in the intervention group versus 39.8% (65/163) in the control group (*P*<.001). Significant differences between the intervention and control groups were found for Pap smear use (*F*_4,1178_; *P*<.001), knowledge scores (*F*_4,1172_=14.946; *P*<.001), attitude scores (*F*_4,1172_=24.417; *P*<.001), and self-efficacy scores (*F*_1,1172_=10.432; *P*<.001).

**Conclusions:**

This study demonstrated that the PSU intervention is effective in increasing the uptake of Pap smear among postnatal women in Seremban district, Malaysia. This intervention module can be tested in other populations of women.

**Trial Registration:**

Thai Clinical Trials Registry TCTR20200205001; https://www.thaiclinicaltrials.org/show/TCTR20200205001

## Introduction

### Background

Cervical cancer is the fourth most common cancer in women worldwide, after breast, lung, and colon cancer [1]. In 2018, new cases of cervical cancer were estimated at 570,000 [1], with varying incidence and mortality rates in developed and developing countries [2]. Nevertheless, cervical cancer is no longer among the 10 most common cancers in women in developed countries, but is now the second most commonly diagnosed and the third leading cause of cancer-related deaths in women in developing countries [3]. A previous study reported that the global incidence and age-standardized rates of cervical cancer were 15.1 per 100,000 and 13.1 per 100,000, respectively, with a mortality rate of 7.4 per 100,000 [4]. Meanwhile, the incidence rate in developing countries ranged from 10.9 (South Asia) to 43.1 (Africa) [5]. The age-adjusted average varied from 12.8 to more than 20 per 100,000 [6], with a mortality rate between 10 and 30 per 100,000 [5]. In Malaysia, cervical cancer is the third commonest cancer among women with an incidence rate of 6.8 per 100,000, an age-standardized incidence rate of 7.6 per 100,00, and a death rate of 5.6 per 100,000 [1].

The Papanicolaou (Pap) smear screening test can be used for the early diagnosis of cervical cancer and it reduces the risk of cervical cancer–related death by 70% [7]. Nonetheless, at least 80% of women in the recommended population groups must undergo Pap smear screening in order for the program to be effective [8]. Postnatal women represent an important population category that may benefit from opportunistic Pap smears screening given that they are within reach of health services [9]. In Malaysia, Pap smear screening and family planning advice are typically recommended to mothers during their postnatal follow-up [10].

### Prior Work

Malaysia’s present Pap smear uptake (PSU) is approximately 47.3% [11], which is lower than the intended goal of attaining 70% coverage [7]. This issue of low PSU persists in Malaysia despite the provision of free Pap smear screening tests that are easily accessible in the country. However, currently the test is performed as part of voluntary or opportunistic screening. Previous studies have demonstrated that low Pap smear screening uptake is associated with poor knowledge [12], negative attitude [13], lack of time [14], lack of family support [15], perception of painful procedure [16], lower economic status [17], and embarrassment [16]. Thus, individual and environmental factors play vital roles in determining Pap smear practice among women in Malaysia. In addition, previous intervention studies mainly used the Health Belief Model, which primarily focuses on individual’s belief [18-21]. Therefore, other theories need to be explored that focus beyond one’s belief. Furthermore, previous studies employed reminder tools such as SMS text messages [22], phone calls [23], and invitation letters [24]. Given that Malaysians are one of the world’s largest WhatsApp users with wide coverage [25], using the WhatsApp group as a follow-up platform could be a useful strategy in an intervention program.

### Objective

This study aimed to examine the impact of a Social Cognitive Theory (SCT)–based intervention and a WhatsApp follow-up measure, namely PSU intervention, to improve the uptake of Pap smear test among postnatal women in Seremban district, Malaysia. It was hypothesized that postnatal women who received the PSU intervention would have higher uptake 12 weeks after the intervention than women who received standard care.

## Methods

### Study Design

A 2-arm, parallel, single-blind cluster randomized controlled study was conducted, which comprised an intervention and a control group. The cluster in this study was defined as a health clinic. The intervention arm received the standard care and intervention package, whereas the control arm received only standard routine care. Standard routine care included brief counseling by health care personnel about Pap smear testing and available brochures. The primary outcome was the PSU, whereas the secondary outcomes were participants’ knowledge, attitude, and self-efficacy on Pap smear.

### Setting and Recruitment

This study was conducted in Seremban, which is the capital city of Negeri Sembilan state in Malaysia with a total population of 620,100 people. Seremban is a developing city that is located about 60 km south of Kuala Lumpur, the capital of Malaysia. It has 12 government health clinics, which are governed by the Seremban Health District Office. This study location was chosen given that the Pap smear screening uptake among women in Seremban district was lower than the national average of 43%, as well as lower than the estimate among postnatal women (39%) [26].

The study population was postnatal women attending Seremban government health clinics. The inclusion criteria were postpartum women who had never participated in Pap smear screening and had a cell phone with WhatsApp installed and internet connection. Meanwhile, the exclusion criteria were postnatal women diagnosed as having cervical cancer, including precancerous stage and postnatal complications, such as postnatal depression, poorly controlled diabetes mellitus, and hypertension. All the aforementioned conditions must have been certified by a medical officer.

### Randomization and Allocation Concealment

The 12 health clinics in Seremban district were randomly allocated into the intervention and control groups at a ratio of 1:1. All the postnatal clinics were number coded, whereas simple randomization was performed using Stat Trek software [27]. During participant recruitment, all participants were informed that an intervention was being offered. Therefore, participants were unaware of group assignment throughout the study. Participants were blinded to the fact that awareness of being part of the control group could influence their responses in the questionnaires. These procedures were conducted by a third party who was not involved in this study. The researcher was only aware of the group allocation after the randomization was performed. Systematic random sampling was employed in selecting participants from each postnatal clinic, and those considered eligible and consented were recruited in the study.

### Sample Size Calculation

The sample size estimation was based on Lemeshow et al [28] sample size determination in health studies. For hypothesis testing, the formula for 2 population proportions was used to compare the 2 groups. The sample size was calculated using the 2 population proportions formula [29], with a power of 80% to detect a true difference and at a 95% CI. Overall, the sample size was computed based on the uptake of Pap smear test [29], with α of .05 and β of .20, an intraclass correlation coefficient of 0.05, an attrition rate of 20%, and an average cluster size of 10 with a design effect of 1.45. The sample size required after adjusting for the clustered design effect was 394, with 197 participants in each arm.

### Intervention

This newly developed intervention module, namely, PSU intervention, used 6 constructs of SCT, comprising cognitive (knowledge), self-efficacy, goal setting, outcome expectation, problem-solving, and reinforcement [30,31]. The module was revised by 2 public health physicians (NA and AB) and 1 family medicine specialist, and the intervention was completed in 2 phases. The first phase was performed via face-to-face and it involved health educational talk and a small group discussion, whereas the second phase entailed a WhatsApp follow-up. This module has been pilot tested among 30 postnatal mothers who are not included in the main study.

This PSU intervention was delivered by ZM who is also a medical doctor. The health educational talk was 15 minutes, which covers the anatomy of female reproductive organs, introduction about cervical cancer, the incidence rate and mortality rate, early diagnostic methods, importance of Pap smear, the positive effects of having Pap smear, and free services available in the government health clinics. This was followed by a 15- to 30-minute small group discussion with approximately 10-15 participants per session. Participants were encouraged to raise any issues or concerns regarding cervical cancer, Pap smear, and any related issues during the face-to-face session. Feeling embarrassed, which was one of the factors that influence PSU, was addressed by using a drape during the screening and this issue was highlighted during the educational talk. It took approximately 30-45 minutes to complete the educational talk and group discussion.

The participants were then recruited in the WhatsApp group for further follow-up and the sessions were conducted weekly for 4 weeks. Allocated time for the WhatsApp group was 1 hour, every Tuesday from 5 to 6 PM. This was the time when the participants were least busy during the week. Nevertheless, participants were also welcome to discuss or ask any questions outside the allocated time. The role of the WhatsApp group was to share information, concerns, and issues; as well as address any misunderstanding on Pap smear and cervical cancer. Besides, it acts as a reminder. The WhatsApp group was made a private group and no other person apart from those recruited by the researcher could access it. No personal information was requested from the WhatsApp group participants and their privacy and confidentiality were protected. Table 1 shows the summary of the contents of educational intervention and WhatsApp follow-up using SCT.

**Table 1:** Summary of health education intervention contents and WhatsApp follow-up using Social Cognitive Theory.

Number	Social Cognitive Theory constructs	Contents	Method
1	Cognitive (Knowledge)	Anatomy of women’s reproductive system Information on cervical cancer Introduction to Pap^a^ smear test Importance of Pap smear test Misperception of Pap smear test	First phase: Educational talk Video on the procedure of a Pap smear test
2	Self-efficacy	List of situations and scenarios related to Pap smear test Ways to overcome the issues	First phase: Group discussion
3	Goal setting	Setting the goal to undergo a Pap smear test Setting the goal to adhere to Pap smear practice	First phase: Educational talk Second phase: WhatsApp group × 4 weeks
4	Outcome expectation	Benefits (positive expectation) of Pap smear test Negative expectations of Pap smear test: embarrassment, discomfort, and minimal pain	First phase: Educational talk Second phase: WhatsApp group × 4 weeks
5	Problem-solving	Problems that might be faced by the participants to undergo Pap smear test	First phase: Group discussion Second phase: WhatsApp group × 4 weeks
6	Reinforcement	Reminders of the importance of Pap smear test and appointment Reminders of usage of drape during the Pap smear test	Second phase: WhatsApp group × 4 weeks

^a^Pap: Papanicolaou.

### Outcomes

#### Primary Outcome

The primary outcome was PSU, which was assessed in the intervention and control groups at 4, 8, and 12 weeks after the intervention.

#### Secondary Outcomes

The secondary outcomes were knowledge, attitude, and self-efficacy assessed immediately and at 4, 8, and 12 weeks after the intervention.

### Instrument

A validated self-administered questionnaire that was divided into 6 sections was employed in this study. The first section focused on the participants’ sociodemographic characteristics, such as birth date, age, ethnicity, educational level, occupation sector, monthly household income, and marital status. Meanwhile, the second section contained 11 questions on knowledge [18] with the 3 options “yes,” “no,” or “not sure.” The third section also consisted of 11 questions on attitude [18], and participants were instructed to select 1 option from a 5-point Likert scale ranging from 1 (strongly disagree) to 5 (strongly agree). The fourth section comprised 14 questions that measured self-efficacy for Pap smear screening, and were evaluated using the Self-Efficacy Scale for Pap Smear Screening Participation (SES-PSSP) Questionnaire [32]. Participants were informed to select only 1 answer from 5 possible options, namely, “definitely,” “very likely,” “probably,” “unlikely,” and “definitely not.” The fifth section explored the participants’ PSU and the option was dichotomous: “yes” or “no.” Participants selecting the “yes” option were further instructed to choose the facilities used in conducting their Pap smear tests.

### Data Collection

The data collection for this study was conducted from February to December 2020. Data were collected at 5 time points: baseline, immediately after the intervention, and at 4, 8, and 12 weeks after the intervention. Because of the COVID-19 pandemic, attendance at the maternal and child health clinics was severely compromised. Data collection using hard copy self-completed questionnaires was switched to Google Forms for the follow-ups at 4, 8, and 12 weeks after the intervention. The link to the Google Forms was disseminated via the WhatsApp group, and participants had 1 week to complete the questionnaire in the Google Forms.

### Data Analysis

All statistical analyses were performed using SPSS (version 25.0; IBM, Inc.). The intention-to-treat principle was used where participants’ data were analyzed based on their initially assigned group. All potential errors were checked prior to data analysis. Descriptive statistics were employed to summarize the data set and the continuous data were assessed for normality. Normally distributed data were presented as mean and SD, whereas median and interquartile ranges (IQR) were used to summarize nonnormally distributed data. Meanwhile, categorical variables were presented in frequencies and percentages.

PSU between the intervention and control groups was compared at each time point using the chi-square test. A generalized linear mixed model was applied to determine the main effects of group, time, and group × time interaction effects for PSU, knowledge, attitude, and self-efficacy between the 2 study groups before and after controlling for covariates. Covariates included were age, ethnicity, education level, and household income. A *P* value of .05 was considered for statistically significant relationships or effects.

### Ethical Approval

This study was approved by the Malaysian Medical Research and Ethics Committee, Ministry of Health (Reference number ID: NMRR-19-2589-50455). During data collection, a written and informed consent was obtained from each of the respondents.

### Data Sharing

All data relevant to the study are included in the article (also see Multimedia Appendix 1).

## Results

### Participants’ Information

**Figure 1:**
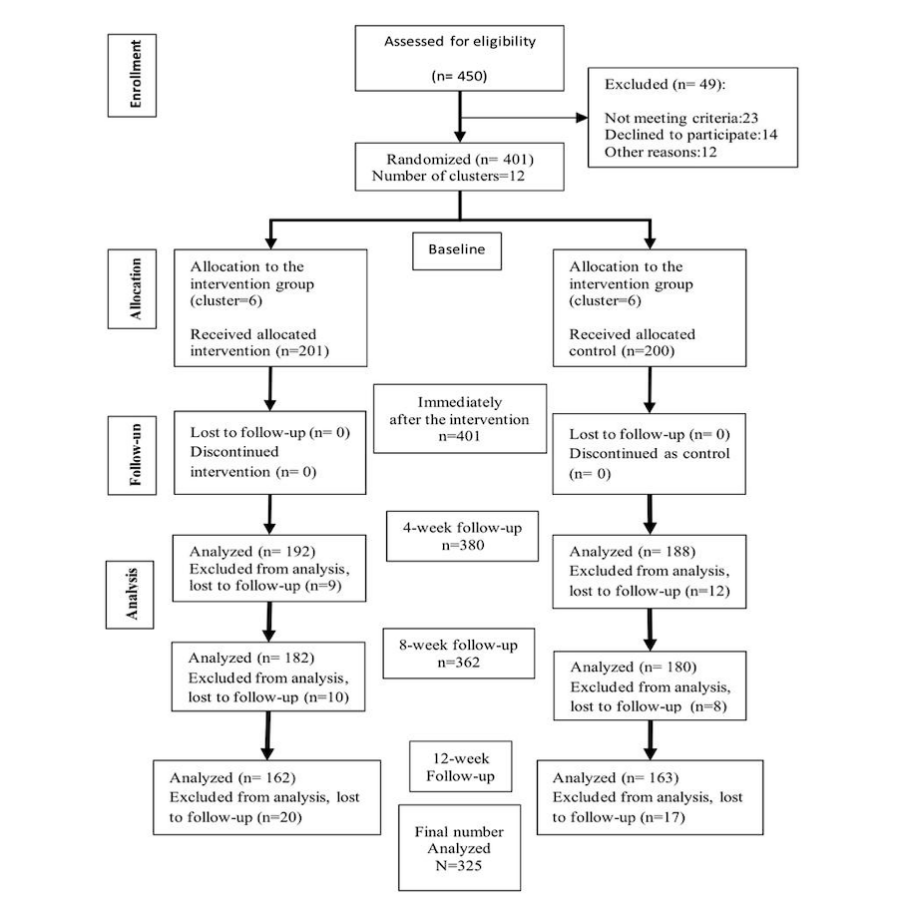
CONSORT-eHEALTH (Consolidated Standards of Reporting Trials of Electronic and Mobile Health Applications and onLine TeleHealth) flowchart [33].

A total of 401 eligible participants (intervention group: n=201, control group: n=200) were recruited in this study. The overall response rate was 81% (325/401) at 12 weeks after the intervention. Figure 1 illustrates the CONSORT-eHEALTH (Consolidated Standards of Reporting Trials of Electronic and Mobile HEalth Applications and onLine TeleHealth) flowchart [33] of the study.

No statistically significant difference was detected between the intervention and control groups at baseline for covariates and outcomes measures (Table 2). A total of 0.26% of the data were missing completely at random (χ^2^_2_=0.867; *P*=.64). Among those who could not be followed up, most were between 26 and 30 years of age, of Malay descent, had a tertiary education, and were in the M40 (RM 2802-RM 5865 [US $634.15-US $1327.37]) income category.

Most participants in both groups were between the ages 26 and 30 years, of Malay ethnicity, married, government servants, and attained tertiary educational level. Household income was categorized into 3 groups: below 40% (B40), middle 40% (M40), and top 20% (T20) of the Negeri Sembilan household income [34]. Most participants were in the M40 category, and 86.5% (347/401) of the participants had Malay ethnicity, followed by Chinese, Indian, and others.

**Table 2:** Participants’ baseline characteristics.

Variables			Intervention (n=201), n (%)	Control (n=200), n (%)	Difference between the conditions	
					Statistical test (*df*)	*P* value
**Age (years)**					7.892^a^ (1)	.25
	20-25		24 (11.9)	19 (9.5)		
	26-30		78 (38.8)	93 (46.5)		
	31-35		53 (26.4)	48 (24.0)		
	>36		46 (22.9)	40 (20.0)		
**Ethnicity**					8.349^a^ (1)	.54
	Malay		178 (88.6)	169 (84.5)		
	Chinese		15 (7.5)	20 (10.0)		
	Indian		8 (4.0)	11 (5.5)		
**Marital status**					—^b^	.43
	Married		197 (98.0)	196 (98.0)		
	Single mother		4 (2.0)	4 (2.0)		
**Level of education**					0.871^a^ (1)	.16
	Secondary		76 (37.8)	68 (34.0)		
	Tertiary		125 (62.2)	132 (66.0)		
**Occupation sector**					0.768^a^ (1)	.18
	None		43 (21.4)	39 (19.5)		
	Government		126 (62.7)	128 (64.0)		
	Private		20 (10.0)	24 (12.0)		
	Self-employed		12 (6.0)	9 (4.5)		
**Household income^c^**					0.768^a^ (1)	.25
		B40 (<RM^d,e^ 2801)	34 (16.9)	32 (16.0)		
		M40 (RM 2802-5865)	122 (60.7)	114 (57.0)		
		T20 (≥RM 5866)	45 (22.4)	54 (27.0)		
Knowledge scores, median (IQR)			8 (3)	6 (4)	1221^f^	.84
Attitude scores, median (IQR)			28 (5)	26 (5)	1090.5^f^	.27
Self-efficacy scores, median (IQR)			38 (8)	36 (7)	1119^f^	.68

^a^Chi-square test.

^b^Fisher exact test.

^c^Household income was categorized into the following based on the Department of Statistics Malaysia: B40, M40, and T20 (specific for Negeri Sembilan).

^d^RM: Malaysian Ringgit.

^e^1 RM=1 US $0.23.

^f^Mann-Whitney *U* test.

### Primary Outcome: Pap Smear Test Uptake

The participants’ PSU at all the time points is presented in Table 3. The results showed that significantly (*P*<.001) more respondents in the intervention group than in the control group had a Pap smear performed before and after controlling for covariates. There was a significant difference in the intervention group at baseline, 4 weeks after the intervention, 8 weeks after the intervention, and 12 weeks after the intervention with *F*_4,1178_=3.222 and *P*<.001.

**Table 3:** Proportion of Papanicolaou smear test uptake among participants at each time point.

Variable	Immediately after the intervention	4 weeks after the intervention	8 weeks after the intervention	12 weeks after the intervention
Intervention, n/N (%)	0/201 (0)	54/193 (27.9)	86/183 (46.9)	110/162 (67.9)
Control, n/N (%)	1/200 (0.5)	24/185 (12.9)	56/181 (30.9)	65/163 (39.8)
Chi-square test	256	54	89	125
*df*	1	1	1	1
*P* value	.66	.04^a^	.02^a^	<.001^a^

^a^Statistically significant.

### Secondary Outcomes: Educational Intervention and WhatsApp Follow-up Outcome

Table 4 presents the generalized linear mixed model results for the total knowledge, attitude, and self-efficacy scores and participants’ intention to adhere to Pap smear practice after controlling for the covariates. The results indicated that significantly more respondents in the intervention group than in the control group had increased their total scores of knowledge (*F*_1,1171_=14.946, *P*<.001), total scores of attitude (*F*_1,1171_=14.946; *P<*.001), and total scores for self-efficacy (*F*_1,1171_=10.432, *P*<.001).

**Table 4:** Effects of educational intervention and WhatsApp follow-up on knowledge, attitude, self-efficacy scores, and intention to adhere to Papanicolaou smear practice among postnatal mothers.

Variables and parameters		*F*	*df1*	*df2*	*P* value^a^
**Knowledge scores**					
	Group	1273	1	1172	<.001^b^
	Time	11.658	1	1172	<.001^b^
	Group × time	14.946	4	1172	<.001^b^
**Attitude scores**					
	Group	458	1	1172	<.001^b^
	Time	35.12	4	1172	<.001^b^
	Group × time	24.417	4	1172	<.001^b^
**Self-efficacy scores**					
	Group	292.038	1	1172	<.001^b^
	Time	13.254	4	1172	<.001^b^
	Group × time	10.432	4	1172	<.001^b^

^a^Using a generalized linear mixed model adjusted for participants’ age, ethnicity, education level, and household income.

^b^Statistically significant.

## Discussion

### Principal Findings

The aim of this study was to evaluate the effects of a PSU intervention based on SCT and using WhatsApp follow-up to improve PSU, knowledge, attitude, and self-efficacy among postpartum women; 12 weeks after the intervention, the intervention group demonstrated a significant increase in PSU. The intervention group also recorded significantly higher knowledge, attitude, and self-efficacy compared with the control group.

Behavioral changes among participants in the intervention group could be attributed to the SCT constructs employed in this study. Given that the health intervention provided a clear picture of cervical cancer and Pap smear test, the participants might have been influenced to have specific goals to undergo the Pap smear test and adhere to Pap smear practice. All the barriers that might arise were discussed comprehensively during the group discussion, which assisted participants in problem-solving and improved their self-efficacy and their PSU. Furthermore, the expected outcome was highlighted during the educational talk and WhatsApp follow-up. Concerns related to the Pap smear test, such as embarrassment, slight discomfort, and minimal pain, were shared with the participants. This information might have motivated the participants to be physically and mentally ready to undergo the Pap smear screening test.

The weekly reminders through the WhatsApp group follow-up reinforced the importance of Pap smear screening and assisted the participants in booking Pap smear screening appointments. Furthermore, the participants were reminded of the usage of drapes and privacy policy during Pap smear test to reduce the feeling of embarrassment. These procedures might have contributed to their positive attitude toward the Pap smear screening test. Self-efficacy is crucial as a determinant of a woman’s decision to uptake the Pap smear. Poor self-efficacy was reported to be influenced by spouses and family members in several developing countries [30]. The SCT constructs used in our study might have contributed to the enhanced self-efficacy observed among participants as depicted in the postintervention self-efficacy scores, which might have encouraged them to undergo the Pap smear screening test.

### Comparison With Prior Literature

The study’s finding on PSU is consistent with a previous randomized control study conducted using SCT in which 70% of participants in the intervention group underwent a Pap smear test [31]. In their study, some of the utilized SCT constructs were cognitive (knowledge), goal setting, and self-efficacy. Nevertheless, the study by Wang et al [31] employed only a face-to-face method, whereas this study utilized the WhatsApp mobile app as an additional follow-up method. Another disparity is the relatively shorter duration in this study compared with 12 months’ follow-up in the previous study [31]. The use of WhatsApp follow-up might assist in reducing the follow-up duration while achieving similar Pap smear screening uptake results. Given the wide acceptance of the WhatsApp platform, this approach could be client-friendly as a follow-up modality that could serve as a reminder and to resolve issues [35].

The improved knowledge scores of participants in this study were consistent with the findings from other interventional studies [36-38]. For instance, participants’ knowledge scores on the importance of Pap smear were significantly impacted following an interactive session in a randomized controlled trial conducted in Korea, which focused on the anatomy of female genitalia and cervical cancer [38]. Another reason for the improved knowledge scores could be due to the participants’ education level. Most participants in this study attained tertiary educational level and their motivation to seek knowledge was higher compared with those with secondary and primary educational levels [39].

### Strengths and Limitations

This is a cluster randomized controlled trial with a good response rate despite the COVID-19 pandemic that occurred during the data collection. A few crucial constructs of SCT were employed as the educational intervention in this study, which was delivered through a face-to-face session and WhatsApp follow-up. This study is among the few local studies investigating the effects of an intervention on PSU, knowledge, attitude, self-efficacy, and intention to adhere to Pap smear practice. To date, this is the first study to employ an educational intervention and a subsequent follow-up technique and reminders for PSU using WhatsApp.

Some limitations of this study include self-reported questionnaires, which may lead to either underreporting or overreporting of results, especially regarding participants’ self-efficacy. This study was conducted in government health clinics, which might have contributed to the low participation of other ethnicities. Specifically, 86.5% (347/401) of the participants were of Malay ethnicity, which was different from the Malaysian demographics pattern of 68.6% [26]. Other women populations were not included in this study as the inclusion criteria entailed postnatal women aged 20-49 years old. Hence, the findings may be different among other nonpostnatal women.

With limited human resources, replicating this intervention might be difficult as it will be an additional burden to the staff at health clinic levels. The WhatsApp group follow-up might be feasible for some health care facilities; however, other centers might find this approach time-consuming and laborious. Factors such as suitability, timing, and human resources need to be considered before implementing an intervention at the clinic level.

### Conclusion

This study suggests that SCT-based health education intervention and WhatsApp group follow-up are effective to improve the PSU among postnatal women, as well as their knowledge, attitude, and self-efficacy. This intervention can be evaluated in other populations that are more representative of Malaysian women and can also be used as baseline data for other intervention studies.

## Appendix

Multimedia Appendix 1 CONSORT-eHEALTH checklist (V 1.6.1).

## Abbreviations

CONSORT-eHEALTH: Consolidated Standards of Reporting Trials of Electronic and Mobile HEalth Applications and onLine TeleHealth
Pap: Papanicolaou
PSU: Pap smear uptake
SCT: Social Cognitive Theory
SES-PSSP: Self-Efficacy Scale for Pap Smear Screening Participation
